# Immuno-reactivity evaluation of Mce-truncated subunit candidate vaccine against *Mycobacterium avium* subspecies *paratuberculosis* challenge in the goat models

**DOI:** 10.1186/s12917-023-03715-z

**Published:** 2023-09-14

**Authors:** Masoud Haghkhah, Zahra Hemati, Abdollah Derakhshandeh, Fatemeh Namazi, Kundan Kumar Chaubey, Shoor Vir Singh

**Affiliations:** 1 Department of Pathobiology, School of Veterinary Medicine, Shiraz University, Shiraz, 71345-1731 Iran; 2 Department of Pathobiology, School of Veterinary Medicine, Shahrekord University, Shahrekord, Iran; 3 Division of Research and Innovation, School of Applied and Life Sciences, Uttaranchal University, Arcadia Grant, P.O. Chandanwari, Premnagar, Dehradun, Uttarakhand, 248007 India; 4 Department of Biotechnology, Institute of Applied Sciences and Humanities, GLA University, Ajhai, Mathura, Uttar Pradesh India

**Keywords:** Control, Immunization, *Mce* gene, Paratuberculosis, Vaccine

## Abstract

**Background:**

Detection of an appropriate antigen with high immunogenicity can be a big step in the production of an effective vaccine for control of Johne’s disease (JD). The aim of this study was to evaluate the efficacy of Mce-truncated protein as a subunit vaccine candidate for the control of JD in experimentally challenged goats.

**Materials and methods:**

Six healthy goat kids were immunized with Mce-truncated protein, and two goats were kept as controls. All kids were twice challenged orally with live *Mycobacterium avium* subspecies *paratuberculosis*(MAP) strain and half the goats from both the categories were sacrificed at 7 and 10 months after start of challenge study. Culture of MAP was performed from all the necropsied tissues to determine the true JD infection status.

**Results:**

Mce-truncated protein only reacted with pooled vaccinated goat sera in western-blot. A significant increase in humoral immune response against Mce protein was also observed in vaccinated goats. Compared to the control group, vaccinated goats gained higher body weights and none of them shed MAP or showed histopatological lesions or colonization of MAP in their necropsy tissues.

**Conclusions:**

The new Mce protein based vaccine provided significant immunity in goats as they could meet the challenge with live MAP bacilli. Although the vaccine used in this study showed the high potential as a new effective vaccine for the control of JD, further validation study is still required to successfully implement the vaccine for JD control program.

**Supplementary Information:**

The online version contains supplementary material available at 10.1186/s12917-023-03715-z.

## Introduction

Johne’s disease (JD) is a granulomatous infection caused by *Mycobacterium avium* subsp. *paratuberculosis* (MAP) an intracellular pathogen that infects domestic ruminants including cows, sheep, goats and buffaloes [1–6]. JD is characterized by gradual weight loss, reduced quality and quantity of milk and other production parameters, emaciation and diarrhea (severe/ continuous/ intermittent) due to the chronic, progressive, granulomatous enteritis and lymph-adenitis [7–12]. However, diarrhea is not a consistent feature in small ruminants [13]. Progression of JD has been often very slow and affected animals do not show clinical symptoms until the final stages of the disease. Because of MAP insidious nature, diagnosis of the infection is very difficult and is often overlooked. Moreover, MAP infections has significant public health concerns duo to its possible involvement and association in human disorders like Crohn’s disease, thyroid and type-1 diabetes [14–19], MAP infected can excrete live MAP into their faeces and milk, so care and attention of farmers during the milking process is very important in order to avoid fecal contamination [20]. MAP bacilli do not get inactivated during pasteurization and may be transmitted to the human population by consumption of contaminated milk and dairy products [18, 21, 22].

Due to the distribution of JD worldwide, economic consequences for the dairy industry and public health concern, suitable JD prevention strategies are needed at priority. After the failure of ‘test and cull’ policy, vaccination is one of the most cost effective control measures for this incurable infectious disease of livestock [5]. Various reports emphasized that vaccination is the best method for control of JD in animals [7, 9, 23]. However, the vaccines developed to date are not fully protective, they are not capable to prevent shedding of MAP in the feces and generate inadequate protection, serious granulomatous reaction and also interfere with tuberculin tests for *Mycobacterium bovis* [7–9, 24]. Inactivated whole-cell and live attenuated MAP vaccines are partially protective and unable to distinguish the antibody response of vaccinated from infected animals [20]. Advances in MAP genome sequencing and molecular biology have provided opportunity to develop several innovative subunits, recombinant or DNA-based vaccines to overcome the drawbacks of the current available JD vaccines [11, 25, 26]. Recombinant subunit vaccines based on single antigen or, protein has been shown to elicit effective systemic and mucosal immune responses against MAP infection [27–29]. The first paratuberculosis subunit vaccine evaluated in cattle was recombinant heat shock protein 70 (Hsp70) with dimethyl dioctadecylammonium adjuvant for subcutaneous administration [30]. Other antigens likely to be included in a subunit vaccine include PPE family proteins (MAP1518 and MAP3184), lipoprotein (LprG and MAP0261c), alkyl hydroperoxide reductases (AhpC, AhpD) and superoxide dismutase, however, these vaccines were not tested as vaccine candidates in the target animals [31]. However, all selected subunit antigens having lack of protective efficacy. To improve this situation, a successful approach might be to focus on cell wall associated or released factors which are necessary for MAP survival in the host. Thus, finding appropriate and unique antigens which do not interfere with tuberculin tests and developing an effective subunit vaccine against JD is required.

Mammalian cell entry (Mce) protein of MAP play an important role in mycobacterial invasion and survival within macrophages [32–36]. These Mce-families of protein are present on the outer membrane of MAP, and are among the virulence-related proteins which can elicit an immune response against mycobacterial infection [22, 33, 36–39]. *MAP2191* gene encodes a protein which is a unique membrane protein of MAP with unknown function [33, 40]. In earlier studies, we have expressed Mce-truncated protein (encoded by a partial part of MAP2191 gene), used as a potential candidate for the development of Mce-ELISA kit for the diagnosis of JD in domestic livestock [16]. In the present study, the protective potential of new Mce-truncated protein based vaccine and immune responses against MAP infection in natural host (goat model) challenged with local pathogenic strains of MAP (named as ZSHU92) were calculated to check the potential of subunit vaccine candidate were used for the effective control of JD.

## Methods

### Ethics approval and consent to participate

This study was approved by the Animal Ethics Committee (AECs) of School of Veterinary Medicine, Shiraz University (permit: 94GCU6M163973) and all methods were carried out in accordance with relevant guidelines and regulations (dated 20 September 2013) and ARRIVE guidelines for reporting animal research as much as possible (https://arriveguidelines.org/). In addition, the informed consent was obtained from the owner of the farm to use animals.

### Animals selection

To study the ‘immune response’ against Mce-truncated protein, 8 apparently healthy (JD negative) male goat kids (≈ 6 weeks, Darabi breed) were selected from a JD free local farm located in Shiraz province, Iran. We have obtained the consent from the owner of the farm to study. These kids were raised under an extensive farming system in a local farm and all the adult goats had no history of paratuberculosis. These goats were shifted to the experimental shed of School of Veterinary Medicine, Shiraz University, Iran for further studies. The physical growth of goats under study and their previous history with respect to symptoms (weakness or diarrhea) of JD were recorded. Three tests (Microscopic, IS*900* PCR of fecal samples and serum ELISA) were used for the detection of MAP infection before the start of actual immunization. All the eight goats under this study had negative status in above three tests and did not show any symptoms of JD, were therefore considered negative for JD and included in this study.

### Antigen selection

According to previous study [16], immunogenic epitopes were predicted over the entire length of the MAP2191 protein. Briefly, *In silico* analysis of the MAP2191 protein was carried out using CLC Genomics Workbench 7.5.1 program (CLC bio, QIAGEN, Germany) and Major histocompatibility complex (MHC) binding peptide prediction algorithms through Immune Epitope Database and Analysis Resource (IEDB-AR) site (http://www.iedb.org/). Predicted B cell epitopes of MAP2191 protein had scores of between 0.5 and 0.9; the minimum prediction cutoff score was set at 0.80. The homology analysis also showed the MAP2191 protein was highly specific as unique to MAP. In addition, the C-terminal portion of MAP2191 protein was more hydrophilic than N-terminal portion and had a high antigenic index as shown in-silico analysis results [22]. Therefore, a new truncated Mce protein coded by the C-terminal (amino acids 154 to 354) portion of this protein that had potential epitopes of T and B cells was expressed, purified using Ni-NTA and confirmed by western blot. A Mce-truncated based ELISA test was developed to evaluate serum samples from ruminant species infected and/or not infected with MAP. In this study, the antigenic potential of the truncated Mce protein coded by selected region of *mce* gene (Accession number: MG754208) was used to develop a new vaccine for the control of MAP infection.

### Assessment of immunization

Purified Mce-truncated protein and booster dose were reconstituted in Freund’s incomplete adjuvant oil and the reconstituted antigen was used to raise the hyper immune serum. At the age of 7 weeks, six of the eight goats (goat numbers 432, 433, 442, 444, 452, 480) were experimentally injected on the left side of neck behind ear with 100 μg/goat of purified Mce-truncated protein, and remaining 2 goats (goat numbers 443 and 453) were kept as a control. One goat of the control group received only 1 ml 1X PBS (goat number 453) and other goat received 500 ul 1X PBS mixed 1:1 (v/v) with the 500 ul Freund’s incomplete adjuvant oil (goat number 443) with the same inoculation schedule. The animals were bled 5 ml blood at 0 days and at every booster and finally hyper immune serum was collected 10–15 days after the last injection (42 days) and were subjected to i-ELISA kit as per Chaubey et al., (2019) to determine the antibody titer against the protein [4]. Inoculation schedule of reconstituted Mce antigen is presented in the Table 1. I-ELISA kit was gifted by the Animal Health Division of Central Institute for Research on Goats, Makhdoom, Po- Farah, Mathura, UP 281,122, India.

**Table 1:** Inoculation schedule of reconstituted Mce protein

Day Trial	Preparation	Quantity of reconstituted antigen	Route of administration
Zero day	2.5 mg purifies protein in 500 UL PBS with 500 ul Freund’s incomplete adjuvant oil	1 ml	500 ul I/P and 500 μl S/C in neck region
21 days (Booster)	2.5 mg antigens in 500 ul PBS with 500 ul Freund’s incomplete adjuvant oil	1 ml	1 ml S/C in neck region
42 days (Booster)		1 ml	1 ml S/Cin neck region
52–58 days		Bleed the animal and harvest the serum	

S/C: subcutaneously, I/P: intraperitoneal

### Immunogenic potential of Mce-truncated protein using western blot analysis

Immunogenicity of the Mce-truncated protein was tested by western blotting using serum samples of immunized goats with Mce-truncated protein based vaccine. Peroxidase-labeled anti-goat whole IgG immunoglobulin (Cat. numbers A8919; Sigma-Aldrich, Inc.) was used as secondary antibody in western blot analysis.

### Challenge strain and inoculum preparation

The strain of MAP used in this study was recovered from an extremely weak and emaciated goat suffering with advances clinical JD at veterinary clinics of Shiraz University. Primary isolation of MAP strain from goat feces was done on Herrold’s egg yolk (HEY) agar, and MAP colonies were identified morphologically, mycobactin J dependency, Ziehl-Neelsen (acid fast) staining of smears and IS*900* PCR and IS*1311* PCR [41]. Methods for fecal decontamination and culture are described in detail in Hemati et al., 2020.

The MAP strain (named as ZSHU92) was isolated from a completely sick clinical animal which had died because of JD. Also, one goat in control group presented the overall pathology and location of JD lesions similar to natural cases in the field. It is confirmed that the MAP inoculum which used is pathogenic and could cause clinical signs of JD. To prepare the challenge inoculum, MAP strain was sub-cultured on 15 HEY agar tubes and was incubated at 37^o^C for further growth and monitored for any extraneous contamination for 10 weeks at 37 °C. One colony of each confirmed MAP growth, which had no colony adventitious organism were transferred to 50 square bottles (25 ml) containing Middlebrook 7H9 broth base supplemented with 1% glycerol, 2 μg/ml mycobactin J (Allied monitor, USA) and 10% OADC for 10 weeks. Another ZN staining and IS*900* PCR assays were conducted on the liquid cultures to confirm the originality of MAP in challenge inoculum. Liquid media were pelleted by centrifugation (2500 g, 15 min, room temperature) in pre-weight falcons (50ml) and the supernatant discarded. After centrifugation, excess fluid of bacterial pellet was drained and accurate bacterial wet weight of each pellet was determined. MAP pellets were suspended in whole pasteurized cow milk to a final concentration of 20 mg MAP per milliliter and mixed by vortex for 4–5 min with four glass beads to break up the MAP clumped bacilli prior to use.

### Experimental infection assessment

**Fig. 1:**
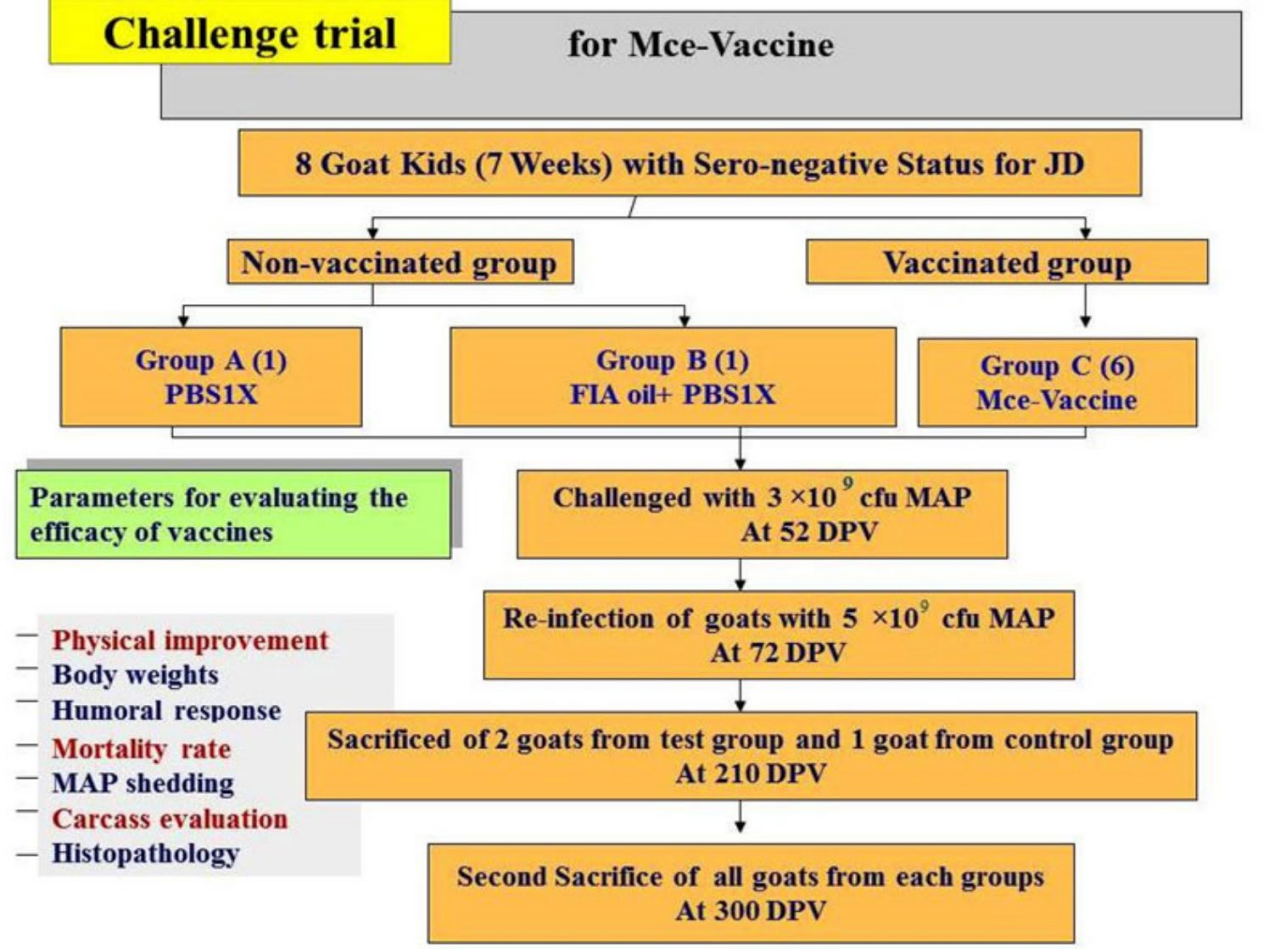
Challenge trial of Mce protein vaccine

To study ‘host response’ against Mce-vaccine was done as per Singh et al., [42], all the goat kids in both immunized and control groups were challenged orally twice at 58 and 72 days post vaccination (DPV) with 15 ml dose (3 × 10^9^ cfu) and 25 ml dose (5 × 10^9^ cfu)in whole cow milk with the homologous strain of the live MAP. Two goats of vaccinated group and one from the control group were sacrificed at 210 DPV and remaining goats were sacrificed at 300 DPV after the 1st challenge (Fig. 1). The challenge inoculum was provided to the kids in a 50-ml sterile syringe which gently pressing on the back of the tongue. Mce-vaccine was evaluated for different parameters (physical condition, fecal shedding of MAP, appearance of MAP DNA in feces and humoral immune response) up to 10 months during progression of JD in experimentally infected goats. Persistent diarrhea, weakness, progressive weight loss, rough skin/ hair and drops in feed conversion efficiency were recorded as major symptoms for declaring the goats as a positive sign of JD symptomatically.

### Monitoring the shedding level of MAP in feces

Fecal samples were collected directly from the rectum of animals in sterilized plastic bags using sterilized plastic gloves and shipped to laboratory with proper details like identification number, photo, and other details of the goats. Approximately, 2 gm of the fecal samples was processed and screened for the detection of MAP by fecal IS*900* PCR [41] and culture for isolation of MAP on HEY medium with mycobactin J [16].

### Humoral immune response by Mce-ELISA and i-ELISA

Blood samples were collected aseptically from the jugular vein of all goats for screening of serum samples by ELISA test. Goats were bled (5 ml blood) at 0 days before experimental infection, two days after each challenge and once every month. In order to measure the induced humoral response, two Mce-ELISA plates were coated with recombinant Mce-truncated protein and i-ELISA kit were coated with semi-purified protoplasmic antigen (sPPA). Test was performed as described in detail in our earlier study [16]. OD values for each serum sample at each sampling time were converted into S/P ratio as described as per Collins [43] and sero-conversation of each individual goat was monitored throughout the study. Positive and negative sera from the confirmed JD infected and uninfected animals were included in each assay as controls.

### Pathological analysis

All experimentally infected goats were euthanized by intravenous injection of sodium pentobarbital at 7 and 10 months after the 1st challenge and necropsied, as described in Fig. 1.To monitor the development of disease, experimentally infected goats were euthanized at 7 and 10 months after the 1st challenge, as described in Fig. 1. Gross lesions of each goat, mainly intestinal thickening, corrugations and enlargement of mesenteric lymph node were recorded. Tissue samples were collected from the ileum, jejunum, and mesenteric lymph nodes (MLN) of each necropsied goat. Tissues were cut, fixed at 10% neutral buffered formalin, embedded in paraffin wax and sectioned at thickening of 5 μm. Duplicate sections stained with hematoxylin and eosin (H&E) and Ziehl-Neelsen (ZN) staining methods were examined by a veterinary pathologist, who was blinded to the groups.

### MAP culture and PCR

Fresh tissue samples from the last portion of mesentery, ileum, the mid portion of jejunum and associated lymph nodes (MLN, ILN and JLN) were separately collected in sterilized bags for acid fast staining, culture on HEY media and molecular detection of MAP by IS*900* PCR.

## Results

### Sero-reactivity of Mce-truncated protein by Mce-ELISA

**Fig. 2:**
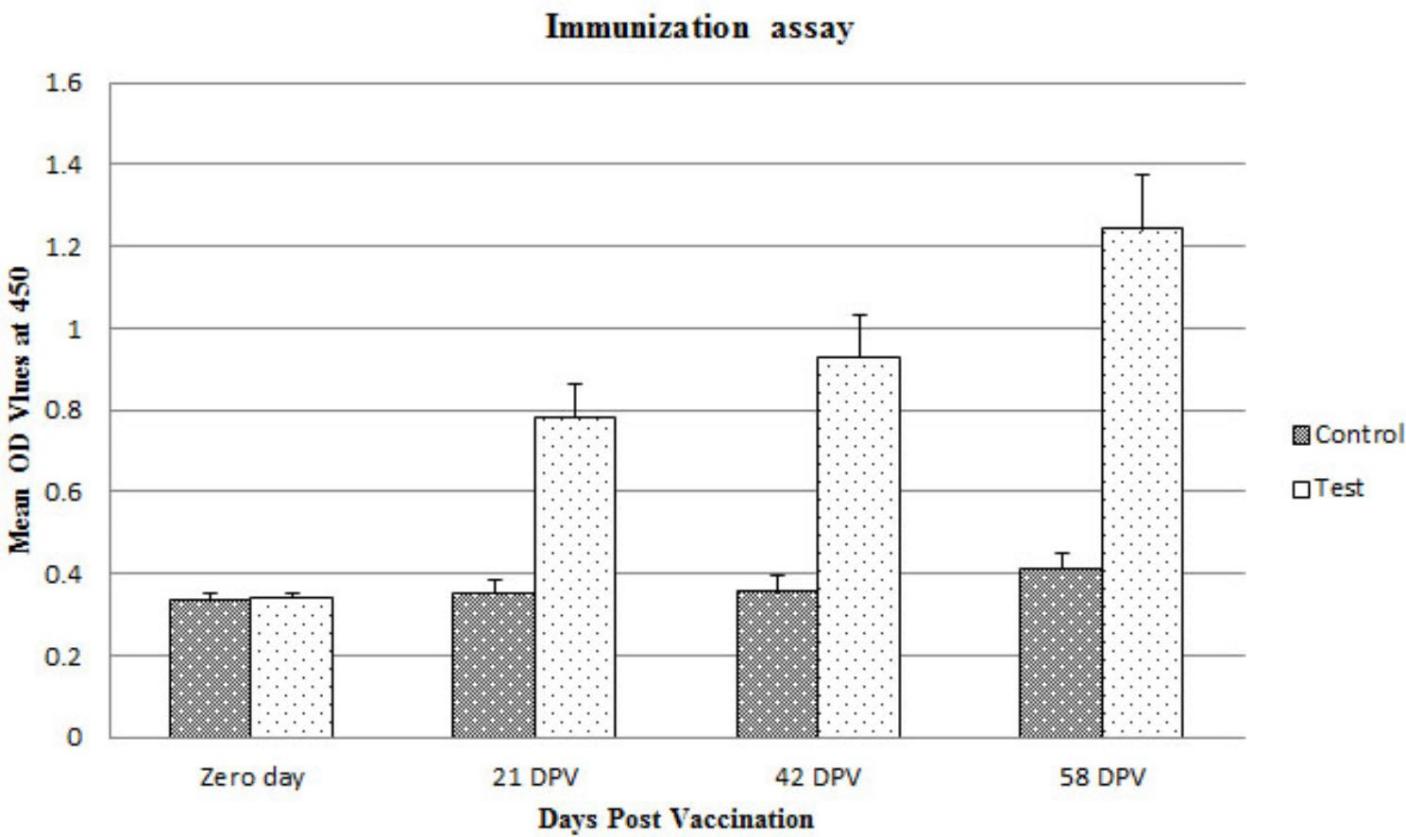
Sero-reaction rates (Mean OD value) in immunized goats and control goats

Serum samples of control group displayed little (Goat number 5, which received only 1X PBS + Freund’s incomplete oil adjuvant) to no reactivity (Goat number 2, which received only 1X PBS) with Mce-truncated protein, while Mce-truncated protein displayed increasing reactivity with immunized goats serum samples from zero day to 58 days post vaccination (Table 2; Fig. 2).

**Table 2:** OD of goat serum samples at different time intervals by Mce-truncated-ELISA

DPV	Animal number							
	480	453	452	444	443	442	433	432
Zero day	0.3	0.296	0.365	0.363	0.338	0.311	0.378	0.354
	0.354	0.326	0.378	0.352	0.37	0.272	0.338	0.35
21 DPV	0.932	0.337	0.435	0.935	0.434	0.734	0.821	0.792
	0.991	0.302	0.36	0.982	0.334	0.812	0.853	0.8
42 DPV	1.151	0.328	0.467	1.07	0.404	0.949	0.923	0.985
	1.14	0.297	0.428	1.077	0.39	0.979	0.988	1.005
58 DPV	1.32	0.401	0.702	1.246	0.47	1.282	1.222	1.526
	1.24	0.343	0.726	1.21	0.428	1.396	1.065	1.961

DPV- Days Post Vaccination

### Western blot analysis of antibody response to Mce-truncated protein

**Fig. 3:**
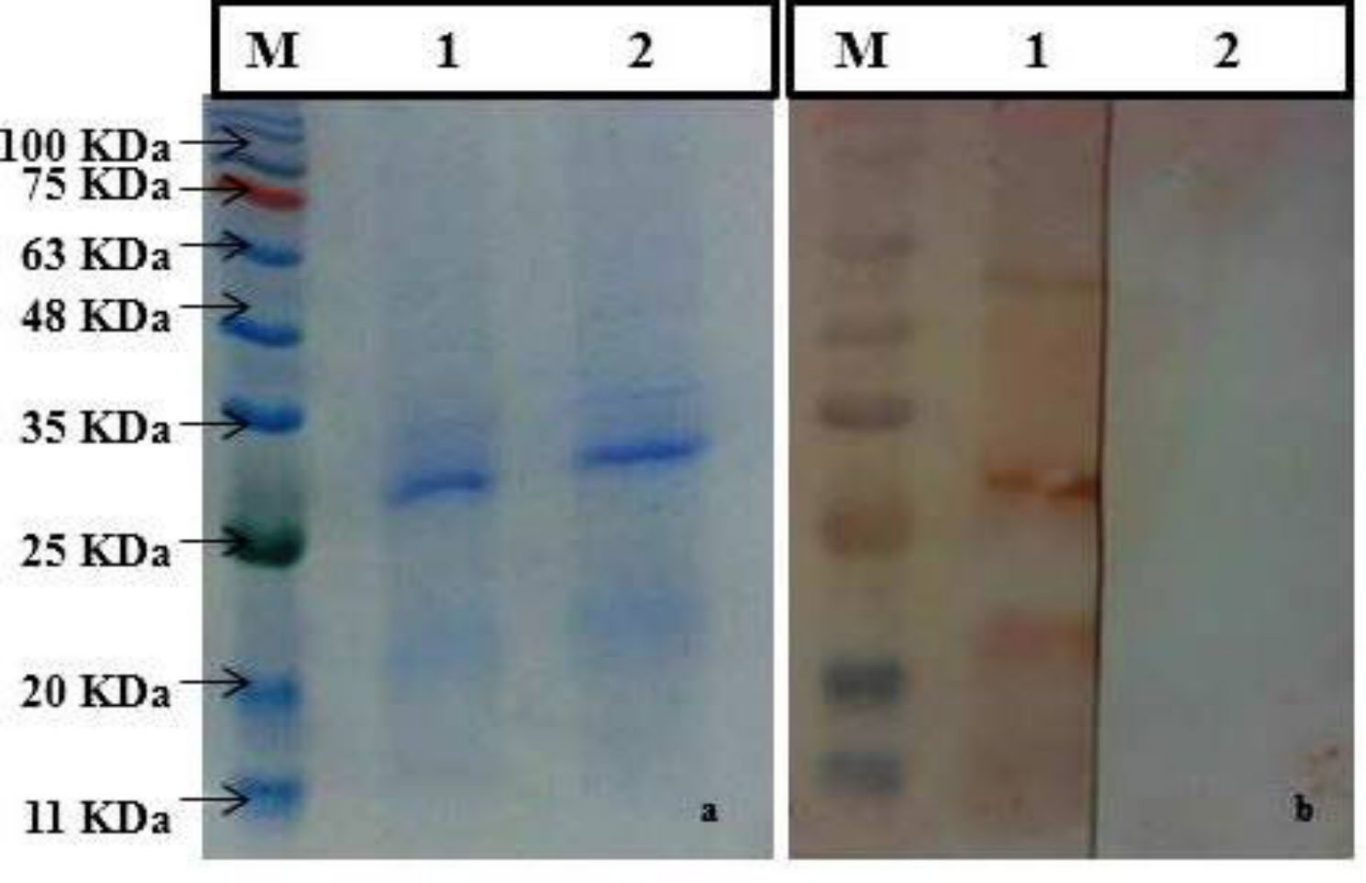
Western blot analysis of AB response to Mce-truncated protein with HRP-conjugated anti-goat/anti-bovine immunoglobulin: (left to right). (**a**) SDS-PAGE analysis, lane M. Protein molecular weight marker (SL7012, CinnaGen). Lanes 1, 2: purified MAP Mce-truncated proteins; (**b**) lane 1: western blot analysis by pooling test goats serum samples, lane 2: western blot analysis by pooling control goats serum samples, lane M: protein molecular weight marker (SL7012, CinnaGen). The blots were cropped

Western blot analysis results using Mce-truncated protein immunized goat serum samples indicated that Mce-truncated protein did not react with pooled serum samples of control goats, but strongly reacted with pooled goat serum samples of challenged goats (Fig. 3).

### Experimental infection and assessment of results

**Fig. 4:**
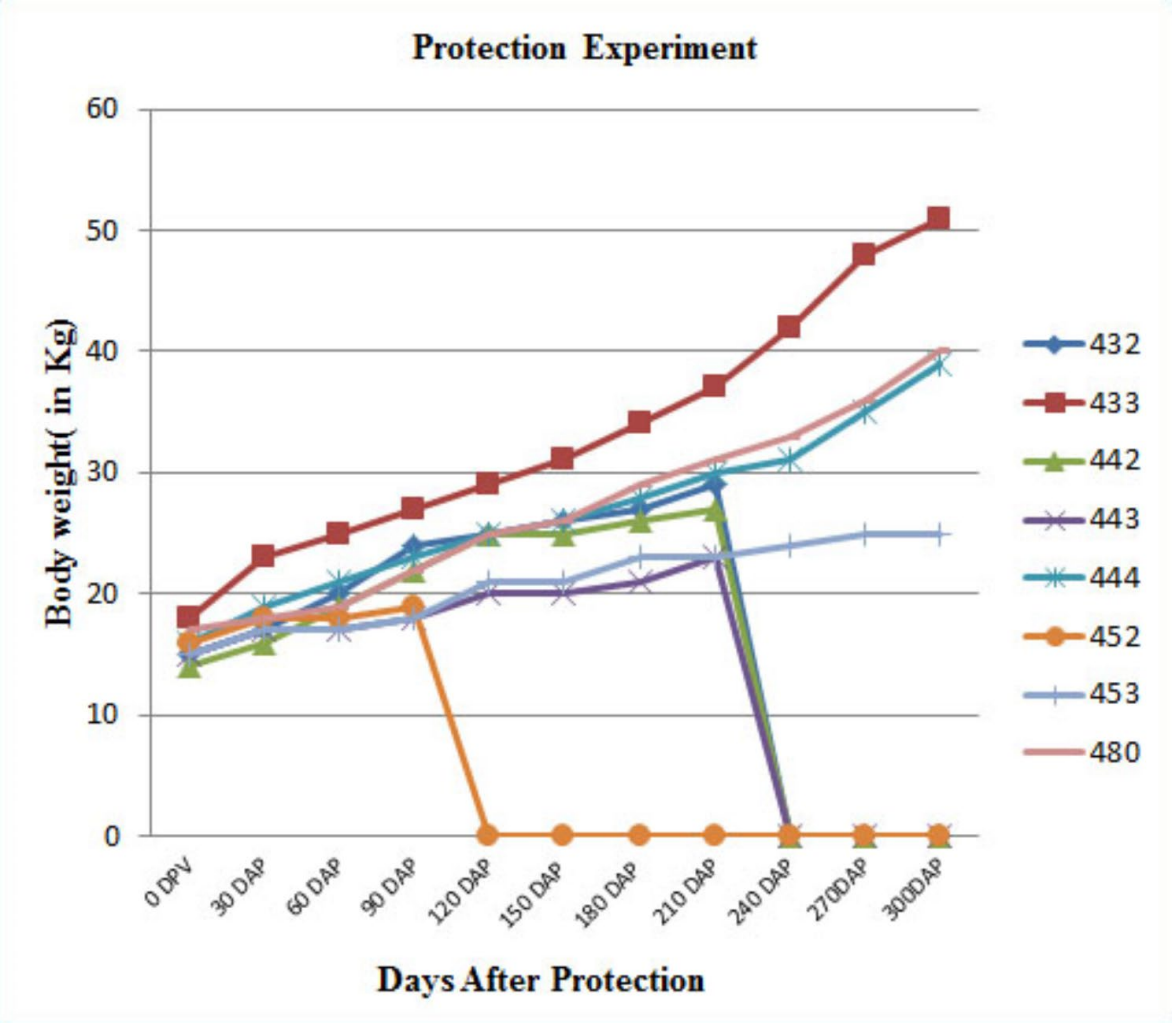
Body weight monitoring. Goat numbers 432, 433 and 442 in the challenged group; Goat number 443 control group sacrificed at 210 DAP; goat numbers 444 and 480 in the challenged group; Goat number 453 in the control group; Goat number 452 in the challenged group died at 90 DAP. Growth rates of the control group (443 and 453) were below the goats in the challenged group

Vaccinated and control goats were normal in condition and did not show clinical symptoms of JD, till 10 months post infection (MPI). However, the appetite of control goats started to decrease with slight deterioration in body weights and physical health from 6 MPI onwards. At 8 MPI gradual weight loss, weakness and deterioration in hair quality and skin pliability were recorded in control goats infected with MAP. Whereas, test goats maintained comparably good health except one goat (goat No, 452), which suffered from pneumonia and died at the 3 MPI without any sign of JD at necropsy. Monitoring of body weight data showed, growth rates of the control group were below the goats in the test group (Fig. 4). However, there was no case of emaciation and cachexia in the test group till 10 MPI, the stage/ level of induced MAP infection was seemed to be late sub-clinical or early clinical JD but not the clinical or late clinical stage of JD in the control group.

### Monitoring of level of MAP shedding via feces

Fecal smears from one of the control goats were only positive for the presence of acid-fast bacilli by ZN- staining at 7 MPI. None of goats from challenged group were shedding MAP during the monitoring period. Based on the number of bacilli in the different observed field, it was concluded that a single control goat, was in the category of low shedder (+/++). Fecal culture results were evaluated weekly up to 16 weeks of incubation. Positive culture was confirmed by microscopy and IS*900* PCR of the colonies. Similarly to microscopy examination, none of the test and control goats shedding were detectable by culture of MAP till 7 MPI. However, at 8 MPI, one of control goats (Goat No. 453) was positive for MAP by IS900 PCR and MAP culture.

### Humoral immune response by Mce-ELISA and i-ELISA

**Fig. 5:**
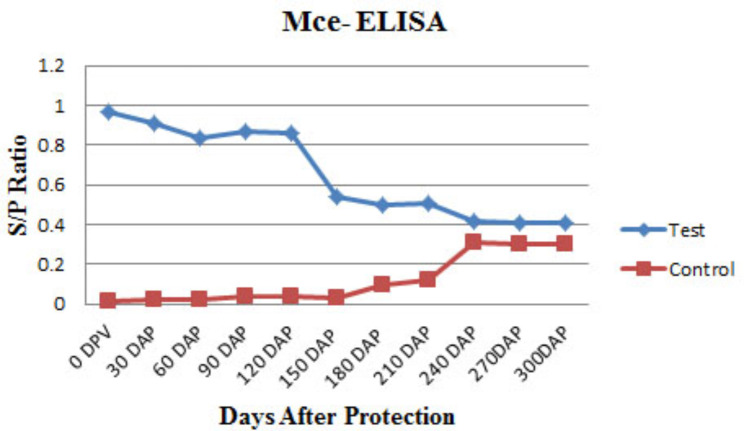
Sero-conversion rates at different time intervals in experimentally infected goats by Mce-ELISA.

**Fig. 6:**
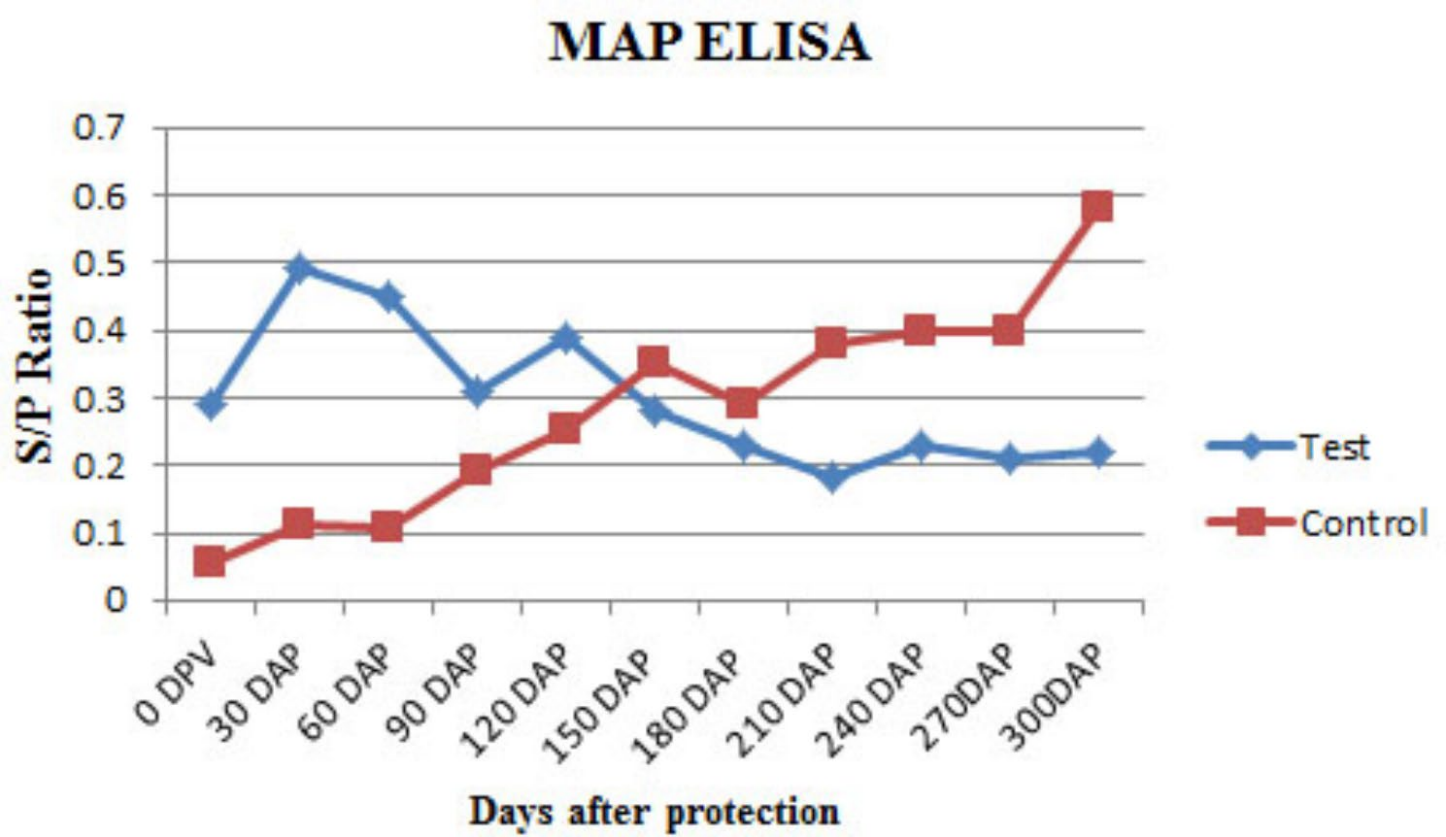
Sero-conversion rates at different time intervals in experimentally infected goats by i-ELISA.

Significant sero-conversion rates were observed in test goats (Figs. 5 and 6), during the monitoring period. There were no strong positive goats in both groups, but on the basis of S/P ratio value, gradual transition of low responding goats to high responding goats started at 6 MPI and onward in the control group with Mce-ELISA (Fig. 5) and i-ELISA (Fig. 6).

### Gross lesions

**Fig. 7:**
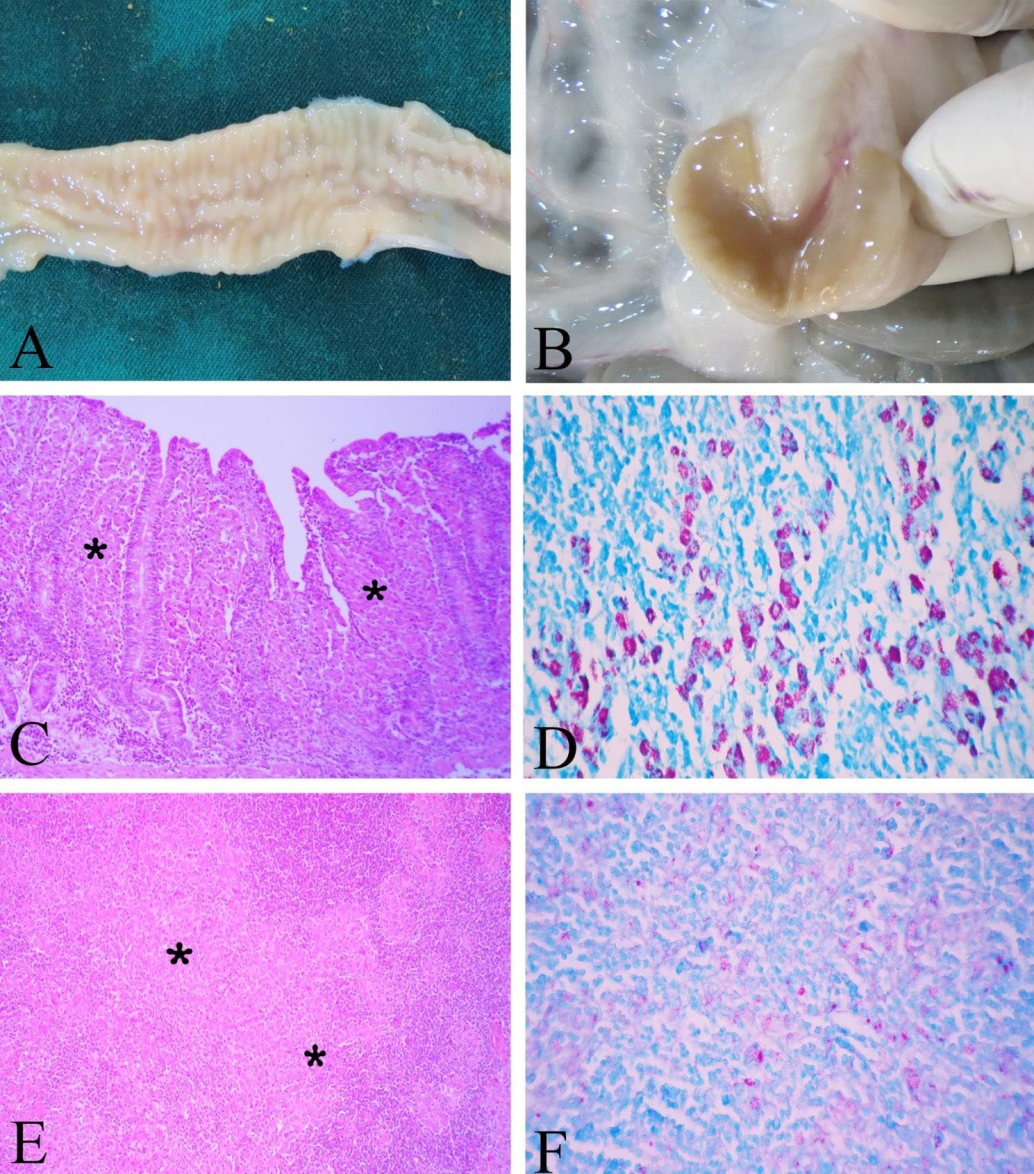
Gross and histopathological lesions of the control group at 10 months post-exposure. (**A**) Intestinal thickening and corrugation was seen; (**B**) lymph nodes were enlarged and edematous; (**C**) Diffuse granulomatous enteritis with infiltration of large numbers of epithelioid macrophages in lamina propria were seen in tissue section (*). H&E, ×100; (**D**) Multibacillary lesion with abundant acid fast bacilli were seen in intestinal section. ZN, ×400; (**E**) Diffuse granulomatous lymphadenitis was seen in tissue section (*). H&E, ×100; (**F**) There were abundant acid fast bacilli in lymph node section, ZN, ×400

At 7 months post-exposure, no special gross lesions were observed in goats of control and vaccinated groups. At 10 months post-exposure, generally comparative evaluation of carcass and fat measurements scored better in vaccinated goats than in the control group. While no lesion was seen in vaccinated goats, the control group showed intestinal thickening and corrugation especially at ileocaecal junction. Enlarged and edematous mesenteric lymph nodes and mild thickened mesenteric lymphatic vessels were observed (Fig. 7A and B).

### Histopathological lesions

At 7 months post-exposure, no histopathological lesions were seen in tissue samples of control and vaccinated groups. Tissue sections were negative in ZN staining at ×1000.

At 10 months post-exposure, tissue sections of control group revealed infiltration of large numbers of epithelioid macrophages in the lamina propria of ileum and jejunum with submucosal lymphangitis, indicating diffuse granulomatous enteritis. Furthermore, diffuse granulomatous lymphadenitis was seen without necrosis and calcification. Multibacillary lesions with abundant acid fast bacilli were observed in lymph node and intestine tissue sections in ZN staining at ×400 (Fig. 7 C-F).

**Fig. 8:**
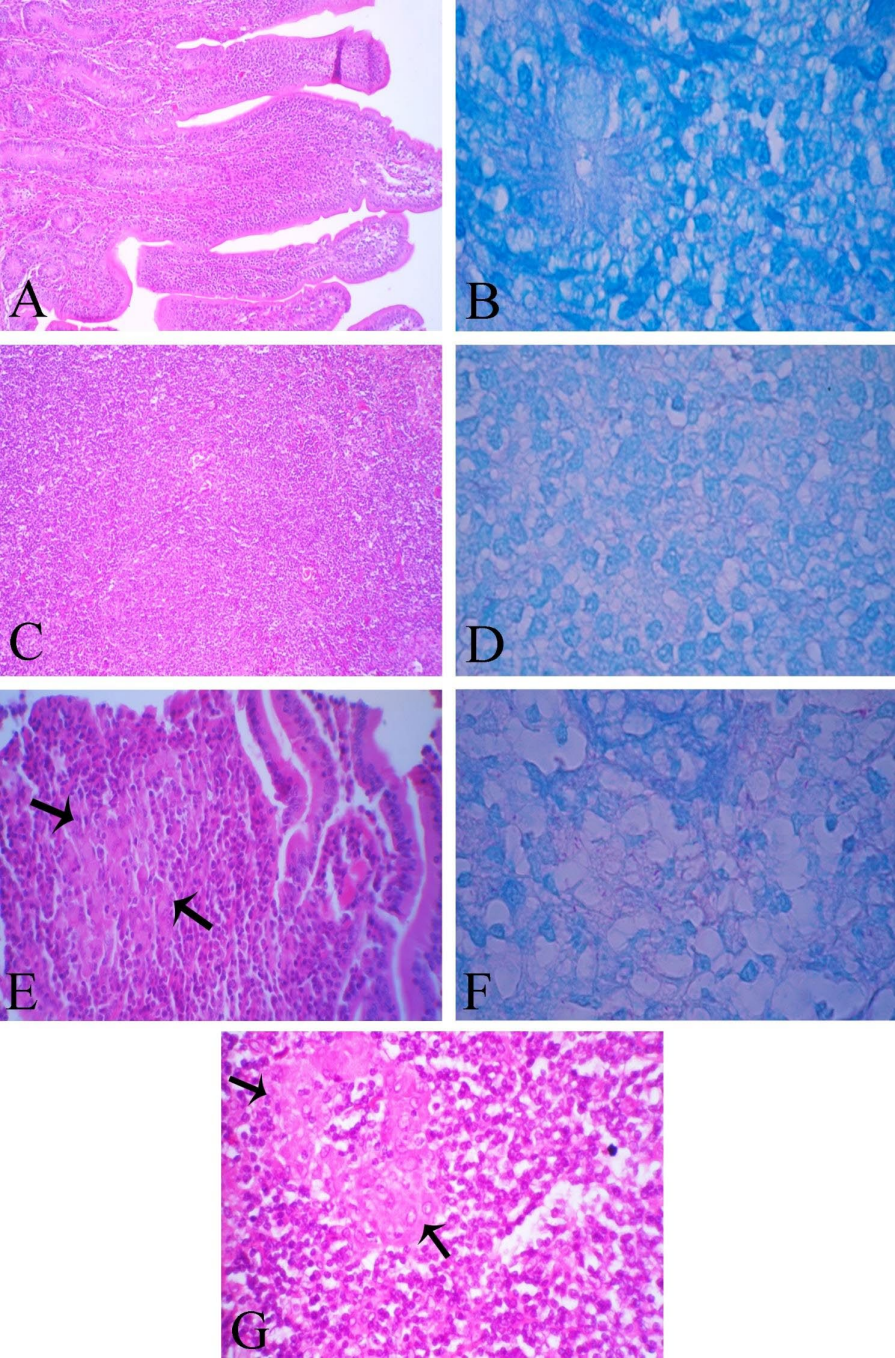
Tissue sections of the vaccinated group at 10 months post-exposure. (**A**) No lesion was seen in the intestinal tissue section. H&E, ×100; (**B**) No acid fast bacilli in intestinal section were observed, ZN, ×1000; (**C**) No lesion was seen in the lymph node section. H&E, ×100; (**D**) No acid fast bacilli in the lymph node section were seen, ZN, ×1000; Except one goat of this group that; (**E**) There were few epithelioid macrophages in the lamina propria (arrows). H&E, ×100; (**F**) Few acid fast bacilli in intestinal section were seen, ZN, ×1000. (**G**) There were few epithelioid macrophages in lymph node section (arrows). H&E, ×400

While no histopathological lesion was seen in tissue sections of vaccinated group and they were negative for ZN staining at ×1000 (Fig. 8A-D), one goat showed diffuse granulomatous enteritis and lymphadenitis with less severity compared to control group, revealing paucibacillary lesion with just few bacilli in ZN staining at ×1000 (Fig. 8E-G).

### MAP culture and PCR

**Fig. 9:**
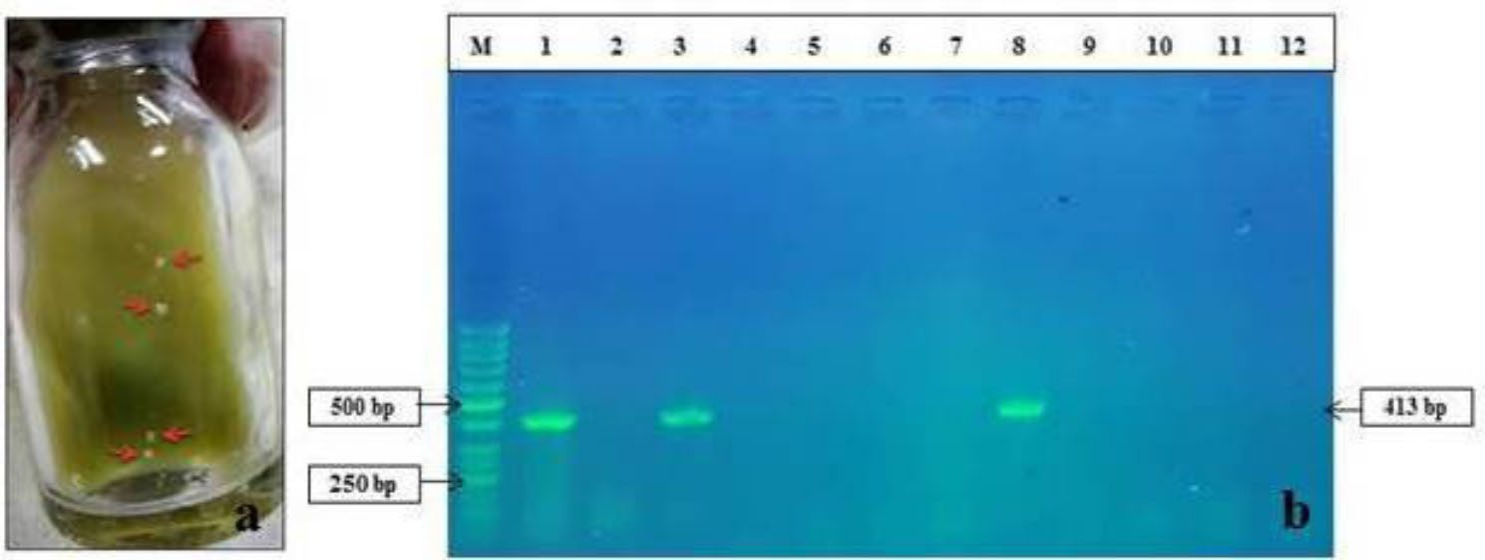
**a**, Characterization of MAP colonies on HEY medium. **b**, Molecular Characterization of the MAP by IS*900* PCR: Lane M- 50 bp Marker (#SM0243, Fermentas), Lane 1: Positive control (MAP DNA); Lane 2: Negative Control (Nuclease Free Water); Lanes 3 and 8: Intestine and MLN of goat number 453; Lanes 4 and 9: Intestine and MLN of goat number 480; Lanes 5 and 10: Intestine and MLN of goat number 432; Lanes 6 and 11: Intestine and MLN of goat number 433; Lanes 7 and 12: Intestine and MLN of goat number 444

Same goats were also positive in tissue culture and PCR (Fig. 9), therefore, confirmed as positive for JD. As shown in Fig. 9b, only samples of intestinal tissue and MLN from a goat (goat number 453) in the control group tested positive for PCR.

## Discussion

Developing the appropriate vaccine for JD is a successful strategy for the treatment of this incurable disease [44]. Current trends in JD monitoring are looking for MAP-specific immunogenic antigens, to develop improved diagnostic and vaccine reagents for animal and human protection [16, 33, 45, 46]. Biological functions of Mce proteins are unknown, but there is growing evidence that *mce* gene expression is important for mycobacterial virulence [47–49]. MAP Mce operon is surface exposed, which corresponds to their proposed roles in pathogenicity of MAP, invasiveness of host cells and interactions between bacteria and hosts, making them suitable candidates for the JD vaccine production [16, 33, 49]. In this study, the immune-reactivity and protective efficacy of the novel Mce-truncated protein to be used as an immunogenic candidate to develop a subunit vaccine against paratuberculosis infection was assessed in the goat models.

*MAP2191* gene codes a protein which is a unique cell membrane (CM) protein of MAP with unknown function [37, 39]. Some studies have looked for unique MAP-specific proteins, which may cause a higher immune reaction in the host [50, 51]. In our previous study, we conducted a full-length epitopes mapping analysis of the MAP2191 protein, which showed that partial sequence (amino acids 154 to 354) of this protein contain most of the dominant epitopes of T and B-lymphocytes and may induce both cellular and humoral immune responses [16, 32]. Analysis of the CLC software also revealed that selected region of the MAP2191 (Mce-truncated) protein was more hydrophilic with a high antigenic index. Panigada et al., [52] have also shown that hydrophilic proteins can represent dominant T-cell epitopes and can be efficiently presented in vitro. Therefore, for this study, Mce-truncated protein was targeted as ‘candidate subunit vaccine’ against JD, since we have assumed that Mce- protein has all the immunogenic traits required to display longer and stronger immune reaction in the host and we have proven this hypothesis to be correct.

In this study, we immunized test goats with the Mce-truncated protein and showed that the control goat serum samples had little or no reactivity with Mce-truncated protein. Whereas, Mce-truncated protein showed growing reactivity with immunized goat serum samples during the monitoring period. These findings were confirmed by western blot assay using same serum samples. This study proved that the truncated Mce protein is immunogenic and capable of producing strong antibody reactions in vaccinated goats. Similar to our findings Ghosh et al., [53] had shown that *PE*, *PPE* and *mce* genes are immunogenic and related to virulence and pathogenesis of mycobacteria. Zhu et al., [54] also identified *mce*, *PE* and *PPE* genes in MAP as markers of virulence.

According to body weight follow-up data after bacterial challenge, goat growth rates in the control group were lower than in the test group. Malone et al., [55] reported changes in body weights in experimentally infected goat kids. They detected major changes in total kids body weight within a week of becoming infected with MAP. The results of their study indicated that JD-infected goat kids had lower gains in body weights in compared to healthy kids of similar age.

Partial or complete reduction of MAP fecal excretion is also an important requirement for an effective JD vaccine [56]. MAP vaccine that minimizes clinical disease and/or fecal excretion would helpful to control JD [24]. It was worth noting that none of the goats in the Mce-vaccinated group were MAP shedder during the entire experiment; whilst the goat No. 453 in the control group was only positive animals by microscopic examination and was multi-bacillary on cultivation of MAP on HEY slants and IS*900* PCR of the growing colonies from fecal samples.

Strong sero-positive goats were also not found in both the groups following challenge with MAP bacilli. Based on S/P ratio values, gradual transition from low-response goats to high response goats began at 6 Months post infection (MPI) and later in the control group with Mce-ELISA and MAP ELISA. These findings indicated that the acquired immune response is related to the JD- compatible lesion development in non-vaccinated goats.

Goats in the vaccinated group had no gross lesions at necropsy. Only one goat of control group (goat No. 453) showed bowel thickening and corrugations, enlarged and oedematous mesenteric lymph nodes and slightly thickened mesenteric lymph vessels at 10 months post-exposure. JD-compatible histopathological lesions were also remarkably observed only in the tissue sections of goat No. 453, indicating diffuse granulomatous enteritis, while the histo-pathological lesions were absent in the other goats. MAP could be cultured from the feces or tissues of a goat that had demonstrated progression of histopathological lesions, which is good evidence for the potential for colonization of our MAP inoculum, therefore, goat confirmed as positive for JD. Immunization with this protein before the MAP challenge provided a high level of protection compared to an unvaccinated control group.

A major problem with the production of experimental JD infections in animal models in previous studies was the absence of clinical symptoms and associated pathology [3, 27, 57]. Faisal and colleagues, (2013b), assessed differential immune response and protection efficiency of a dampened strain of Salmonella expressed partial 74 F fragment of the MAP protein as a provocative vaccine in a goat model [27]. Their data showed that the Sal-74 F vaccine was unable to significantly improve its protective effectiveness and has not restricted MAP colonization of infected goats. In their study, no histopathological lesions were seen in any of the tested tissues in both control and vaccination groups, probably because the goat kids were kept only for a short time (6 months) after the MAP challenge. Some studies have recommended standardized experimental MAP inoculation systems in which the dose and strain of MAP are set for an infection resulting reproducible and as close as possible to a natural infection [3, 58]. In the context of our work, JD-compatible lesions were not seen in Mce-vaccinated goats and only one of the control goats showed mild clinical signs and was positive for MAP in fecal and tissue culture and histopathology. This goat replicated the overall pathology and location of JD lesions similar to natural cases in the field. It confirmed that the MAP inoculum which used in this study could cause clinical signs and that our subunit vaccine could prevent the disease in the Mce immunized group within a similar time frame.

Multiple MAP proteins have been identified to induce various degrees of protection against experimental JD infection [27, 58–61]. In a study by Chen et al., [25], a new 74 kDa fusion protein (Map74F) was produced and tested for efficacy in the mouse. The results of their study showed that the recombinant Map74F protein was protective and able to induce a good humoral and cellular immunity in vaccinated mice. Kathaperumal et al., [9] goat kids were vaccinated with four antigens (Ag85A, Ag85B, SOD and MAP74F) with or without adjuvant DDA and were challenged with MAP. Their subunit vaccine elicited a good Th1 response and provided protection from MAP infection in the goat model.

However, the sample size, particularly in the control group of this study, limits the efficacy of the study in reaching a firm conclusion. We feel that subunit vaccines are the most promising ways for a successful vaccination strategy. Such vaccines need to be co-administered with appropriate adjuvants to raise a strong as well as protective immune response. No histopathological lesions were observed in any of the tissues examined in the vaccination groups, probably because these animals were only kept for a short time (10 months) after the challenge. It would have been interesting to see if lesions developed later if we had been able to hold these animals longer. Therefore, in order to make the JD control program effective, it is essential that this antigen be tested in a wider animal group for a longer time.

In conclusion, ability to reach high levels of MAP-specific antibodies and reduce histo-pathologic lesions and reduced burden of MAP in fecal and tissue samples of challenged goats, suggest that the specific MAP2191 protein has antigenic properties and potential to use as vaccine that may prevent the vaccinated goats from becoming infected. These findings may be useful to support this candidate antigen as an effective subunit vaccine for JD control programs, further studies also needed for validation.

### Electronic supplementary material

Below is the link to the electronic supplementary material.

Supplementary Material 1

## Data Availability

The data sets used and/or analyzed during the current study are available from the corresponding author on reasonable request.
