# Supplementary figures and images for: Immuno-reactivity evaluation of Mce-truncated subunit candidate vaccine against Mycobacterium avium subspecies paratuberculosis challenge in the goat models
Source: BMC Vet Res. 2023 Sep 14;19:157. doi: 10.1186/s12917-023-03715-z (PMC10500891; doi:10.1186/s12917-023-03715-z)

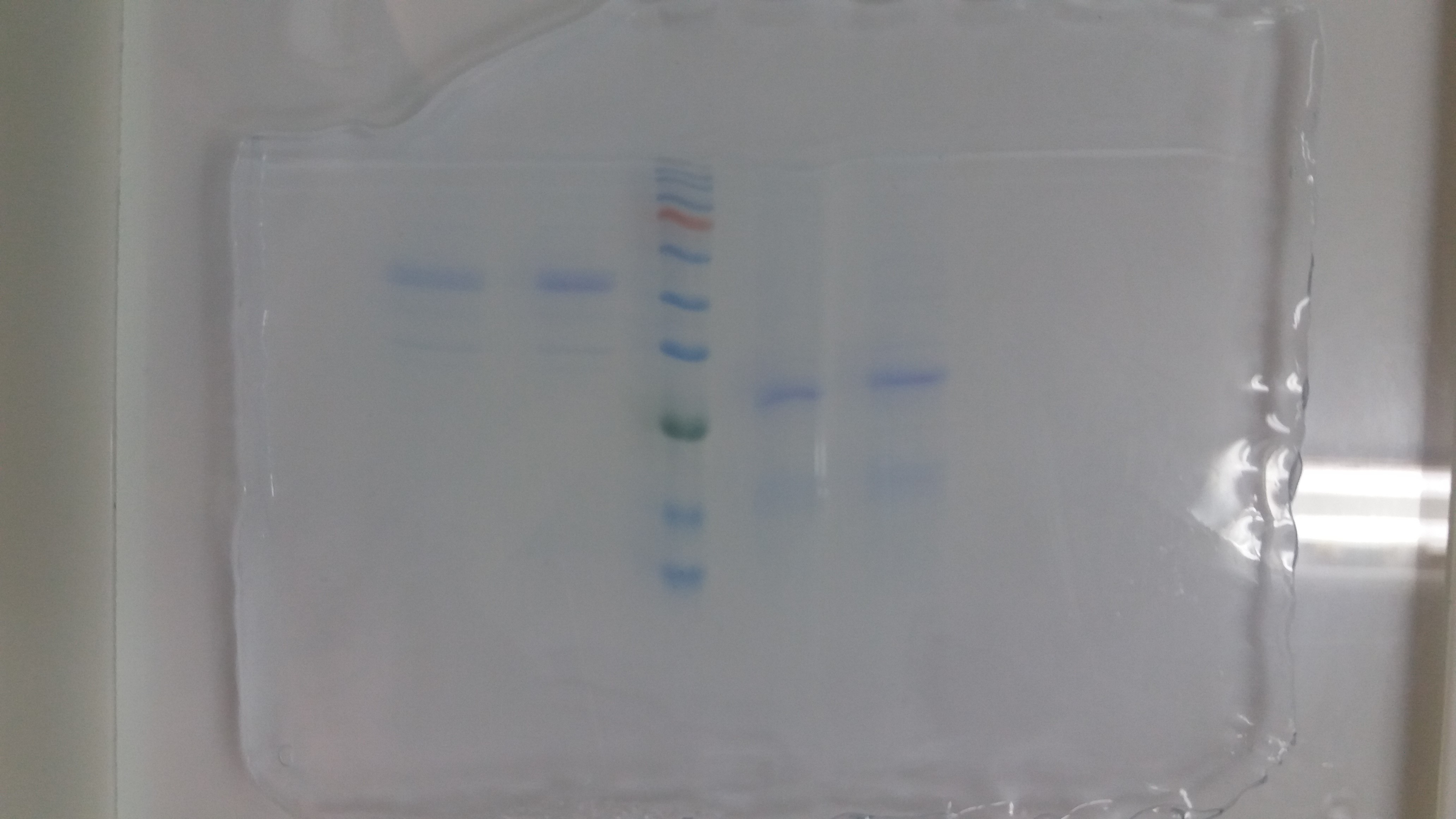


M 1 2

Fig 3A


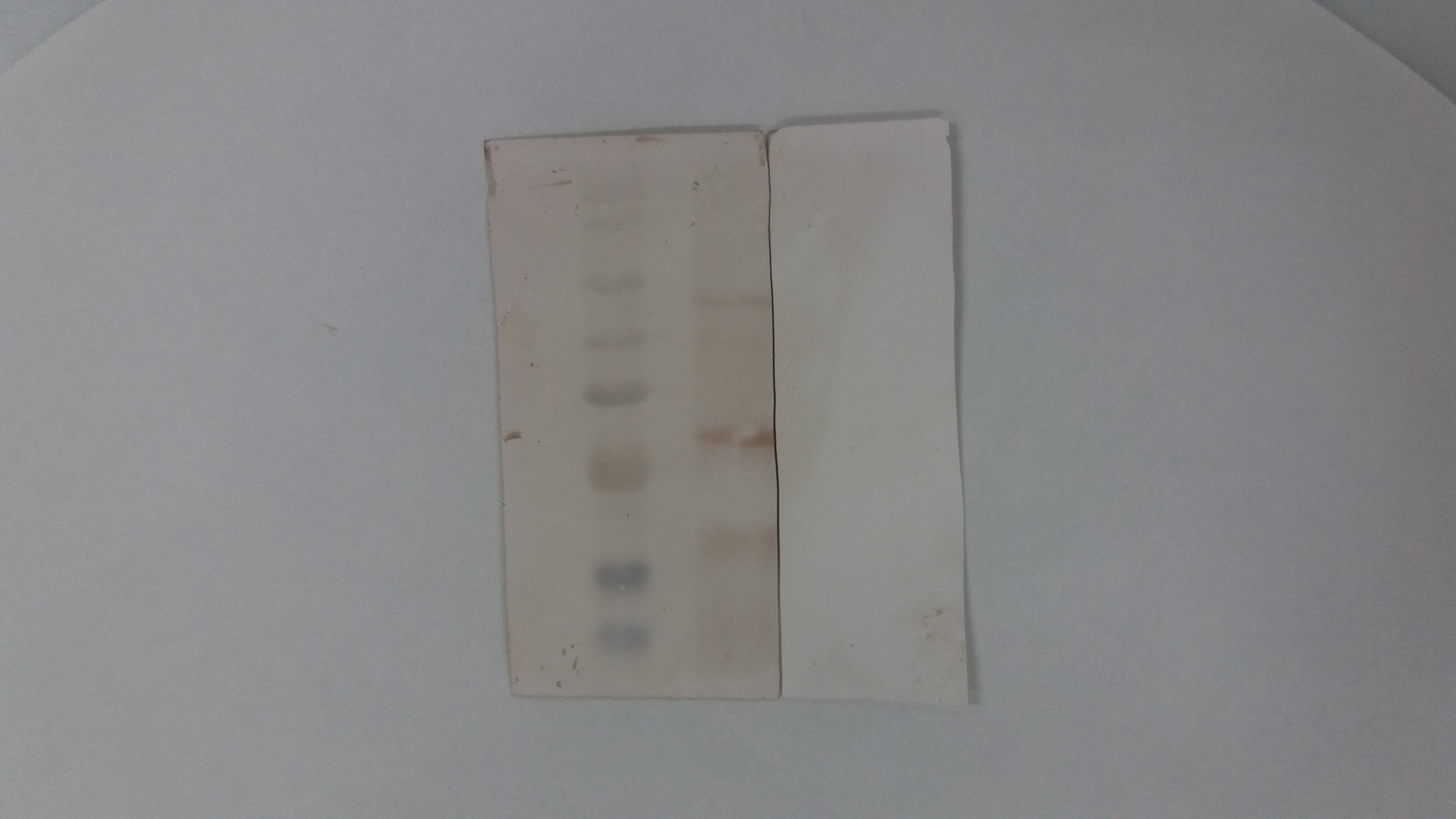


M 1 2

Fig 3B


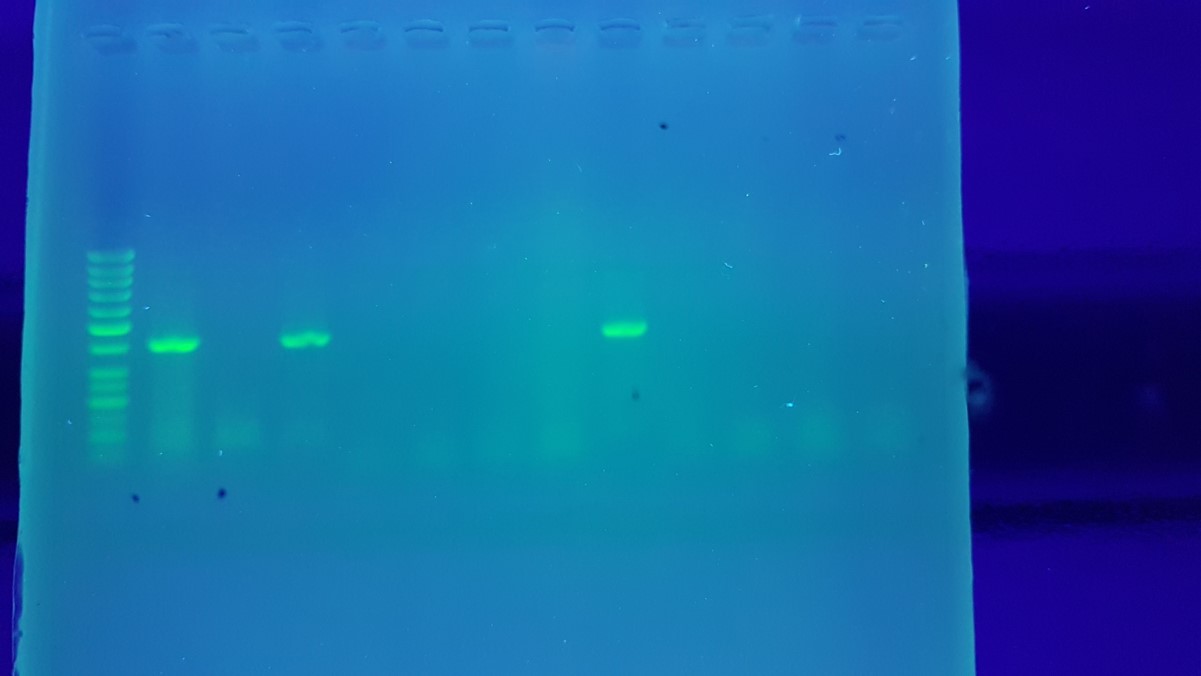


Fig: 9

Supplement: Supplementary file 1 — Supplementary Material 1 [file 12917_2023_3715_MOESM1_ESM.doc]
